# Supplementary material for: Comparison of Primer-Probe Sets among Different Master Mixes for Laboratory Screening of Severe Acute Respiratory Syndrome Coronavirus 2 (SARS-CoV-2)
Source: Biomed Res Int. 2020 Sep 25;2020:7610678. doi: 10.1155/2020/7610678 (PMC7528141; doi:10.1155/2020/7610678)
Supplement: Supplementary Materials — Supplementary Table: information of primers and probes were used in this study. [file 7610678.f1.docx]

Supplementary table. Information of primers and probes were used in this study.

| No. | Primer/Probe | Country | Type | Sequence (5’ to 3’) | Position | Target |
| --- | --- | --- | --- | --- | --- | --- |
| 1 | IDT | US | F1 | ACAGGTACGTTAATAGTTAATAGCGT | 26,141-26,253 | E |
| 2 | Phu Sa | Vietnam | R2 | ATATTGCAGCAGTACGCACACA |  |  |
| 3 | Tib-Molbiol | Germany | P1 | FAM-ACACTAGCCATCCTTACTGCGCTTCGBBQ |  |  |
